# Assessment of the Prioritization of Diagnostics in National Action Plans for Antimicrobial Resistance

**DOI:** 10.1093/cid/ciag020

**Published:** 2026-04-29

**Authors:** Georgia Lamb, Scott J C Pallett, Esmita Charani, Emmanuel Marx Kanu, Saleh A Alqahtani, Maria Virginia Villegas, Luke S P Moore

**Affiliations:** Division of Infection, Barts Health NHS Trust, Royal London Hospital, London, United Kingdom; Clinical Infection Department, Chelsea and Westminster Hospital, London, United Kingdom; Centre of Defence Pathology, Royal Centre for Defence Medicine, Queen Elizabeth Hospital, Birmingham, United Kingdom; Division of Infectious Diseases and HIV Medicine, Department of Medicine, Groote Schuur Hospital, University of Cape Town, Cape Town, South Africa; Masanga Medical Research Unit, Masanga Hospital, Masanga, Sierra Leone; Centre for Tropical Medicine and Travel Medicine, Department of Infectious Diseases, Amsterdam University Medical Centres, University of Amsterdam, Amsterdam, The Netherlands; Division of Gastroenterology and Hepatology, Weill Cornell Medicine, NewYork, New York, USA; Liver, Digestive, and Lifestyle Health Research Section, and Organ Transplant Centre of Excellence, King Faisal Specialist Hospital and Research Centre, Riyadh, Saudi Arabia; Grupo de Resistencia Antimicrobiana y Epidemiología Hospitalaria, Universidad El Bosque, Bogotá, Colombia; Clinical Infection Department, Chelsea and Westminster Hospital, London, United Kingdom; Infection and Immunity Department, North West London Pathology, London, United Kingdom; National Institute for Health Research Health Protection Imperial Biomedical Research Centre, Imperial College London, Hammersmith Campus, London, United Kingdom

**Keywords:** point-of-care, POC, diagnostics, antimicrobial stewardship

## Abstract

**Background:**

Clinically diagnosing antimicrobial resistance (AMR) remains a challenge, with significant variation in countries’ readiness and ability to address it. This study explores how diagnostic capabilities feature in national action plans (NAPs) for AMR, to respond to and manage infections in human health.

**Methods:**

A targeted analysis was conducted of available NAPs of World Health Organization member states. NAPs were analyzed using predefined search terms and an analytical framework for diagnostic modality, key diagnostic activities (laboratory capacity, external quality assessment, surveillance, antimicrobial stewardship, and research) and key performance indicators (KPIs) specific to diagnostics.

**Results:**

Of the 142 NAPs analyzed, 136/142 (95.8%) included diagnostics, mostly in the context of surveillance (131/142 [92.3%]) and building laboratory capacity (113/142 [79.6%]). Culture-based diagnostics appeared in 124/142 (87.3%) NAPs, point-of-care (POC) diagnostics in 47/142 (33.1%), and laboratory-based molecular diagnostics in 47/142 (33.1%). POC diagnostics were included most frequently in high-income country (HIC) NAPs (24/44 [54.5%]), and NAPs of the European region (21/37 [56.8%]). Diagnostic-specific KPIs were found in 35/142 (24.6%) NAPs, and KPIs specific to POC diagnostics in 9/142 (6.3%). Diagnostic KPIs were included most frequently in NAPs of the African Region (15/36 [41.7%]) and low- and middle-income countries (13/42 [31.0%]).

**Conclusions:**

While diagnostics feature in most NAPs, POC diagnostics are underrepresented, particularly in low- and middle-income countries lacking diagnostic infrastructure and resources. The lack of KPIs, particularly in HICs, prevents effective evaluation. This study highlights the need for improved strategic planning to ensure NAP objectives are translated into real-world deliverable action to manage AMR.

## Teaser

We reviewed national action plans (NAPs) for antimicrobial resistance (AMR) for prioritization of diagnostics to manage AMR in human health. Diagnostics feature in 96% of NAPs, but only 25% of NAPs feature diagnostic-specific key performance indicators, with point-of-care diagnostics underrepresented.

**(See the Editorial Commentary by Mo on pages e237–9.)**

Considerable efforts have been made to support countries’ capacity to respond to and manage the threat of antimicrobial resistance (AMR). Despite this, AMR continues to emerge as a critical risk to global public health [1]. The 2015 United Nations (UN) High-Level Meeting on AMR empowered the World Health Organization (WHO) to develop a global action plan (GAP), requiring member states to develop individual national action plans (NAPs), but this has been met over the last decade with variable response [2, 3]. More recently, the 2024 UN High-Level Meeting on AMR invoked a greater focus on optimizing use of diagnostics to stem AMR [4], including commitments to ensure access to reliable testing and investment in both the development and implementation of “innovative, rapid, effective, validated, and affordable diagnostics and laboratory systems.”

Marked variation exists between countries’ capability and capacity to use diagnostics to support AMR surveillance and individual patient management [5]. This is particularly true in low-income countries where there remains a considerable need to upgrade AMR surveillance infrastructure [2, 6]. Microbiological culture can be limited or unfeasible in resource-limited settings [7], and point-of-care (POC) diagnostics may offer rapid, more cost-effective alternatives [8, 9].

Variation in political commitment, resource constraints, laboratory infrastructure and information systems, and governance between countries is a considerable challenge in developing an effective and coordinated international AMR strategy [3]. NAPs (where available) are likely to vary therefore in strategy and priorities, including for AMR diagnostics. With technology rapidly evolving, and in the context of the revised UN AMR statement, it remains unclear how aligned the GAP and NAPs are to the need to integrate diagnostics into the approach to combat AMR [10]. At this crucial time where most countries with published NAPs are considering a second or third iteration, we have undertaken a targeted literature review and critical analysis of existing NAPs to assess current prioritization of diagnostics for managing AMR in human health.

## METHODS

### NAP Search and Selection Strategy

We conducted a targeted literature review and critical analysis of the publicly available NAPs of WHO-recognized member states. NAPs were retrieved from the WHO library of NAPs where available [11]. An additional search was performed to identify NAPs available in the UN Food and Agriculture Organization legal database [12] and/or national online health ministry repositories using the terms “national action plan,” “NAP,” “antimicrobial resistance,” “AMR,” “strategy,” and the country of interest. NAPs written in English and French were analyzed by co-authors (G. L. and S. J. C. P.) as written. For NAPs where an English or French version was unavailable, DeepL machine translation was used [13]. The search was performed in April 2025. Where several NAP iterations were available, the most recent version was used for analysis. Identified NAPs were classified according to WHO region and to World Bank income index classification (high-income country [HIC], upper-middle-income country [UMIC], lower-middle-income country [LMIC], and low-income country [LIC]) [14]. The Americas were further categorized into North America and Latin America and the Caribbean (LAC)—subregions with independent governance and AMR surveillance networks.

### Data Extraction and Analysis

Each NAP underwent manual line-by-line evaluation using a predefined, analytical framework to identify and categorize key themes around diagnostics within NAPs.

Review of the WHO GAP shaped diagnostic themes for analysis. Three diagnostic modalities were focused on: (1) culture-based diagnostics including antimicrobial susceptibility testing (AST); (2) POC diagnostics, including rapid diagnostic tests (RDTs) intended for use at the POC, for example, rapid antigen tests and POC nucleic acid tests such as Gene Xpert; and (3) laboratory-based molecular diagnostics, for example, whole genome sequencing (WGS). Five key domains for diagnostics-related activities emerged, derived from the WHO's Antimicrobial Resistance Diagnostic Initiative [15] and Resolution WHA76.5: Strengthening Diagnostics Capacity [16]: (1) building laboratory capacity, (2) external quality assessment (EQA) of diagnostics, (3) diagnostics for AMR surveillance at a population level, (4) diagnostics for antimicrobial stewardship (AMS) at the individual patient level, and (5) research and development (R&D) for diagnostics. Finally, NAPs were assessed for inclusion of key performance indicators (KPIs) specific to diagnostics. These were defined by the presence of a measurable, objective metric, with a specified target and timeframe. Data against these criteria were extracted for comparison according to WHO region and to World Bank income index classification [14]. Following this, a qualitative summary, using illustrative examples from reviewed NAPs, were provided for each theme. Microsoft Excel was used to store extracted data and perform descriptive analysis of the data.

This study was an evidence synthesis of previously published documents; as such, no ethical approval was required.

## RESULTS

**Figure 1.**
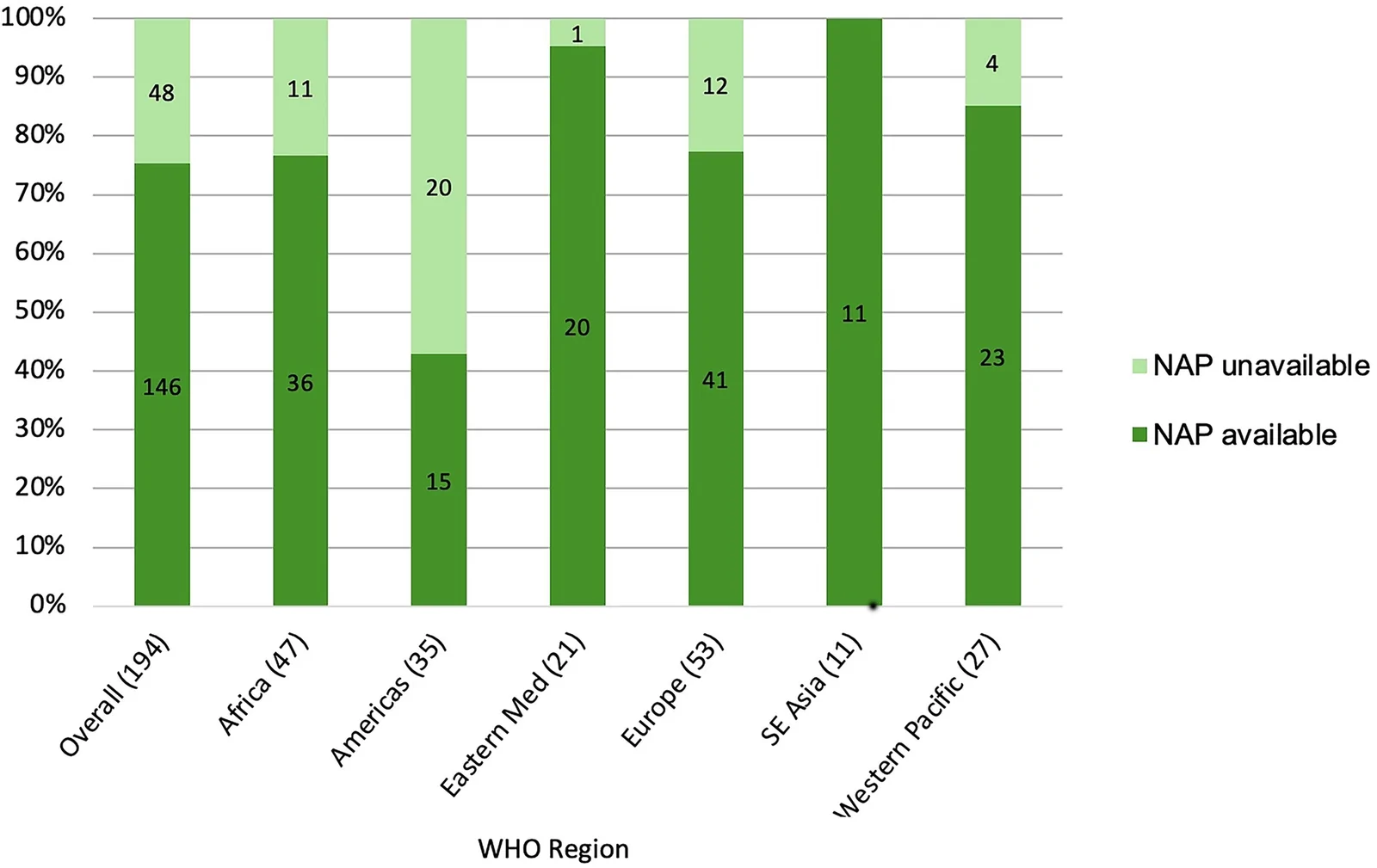
Availability of national action plans on antimicrobial resistance by World Health Organization region, as of April 2025. Abbreviations: Eastern Med, Eastern Mediterranean; NAPs, national action plans; SE Asia, South-East Asia; WHO, World Health Organization.

**Figure 2.**
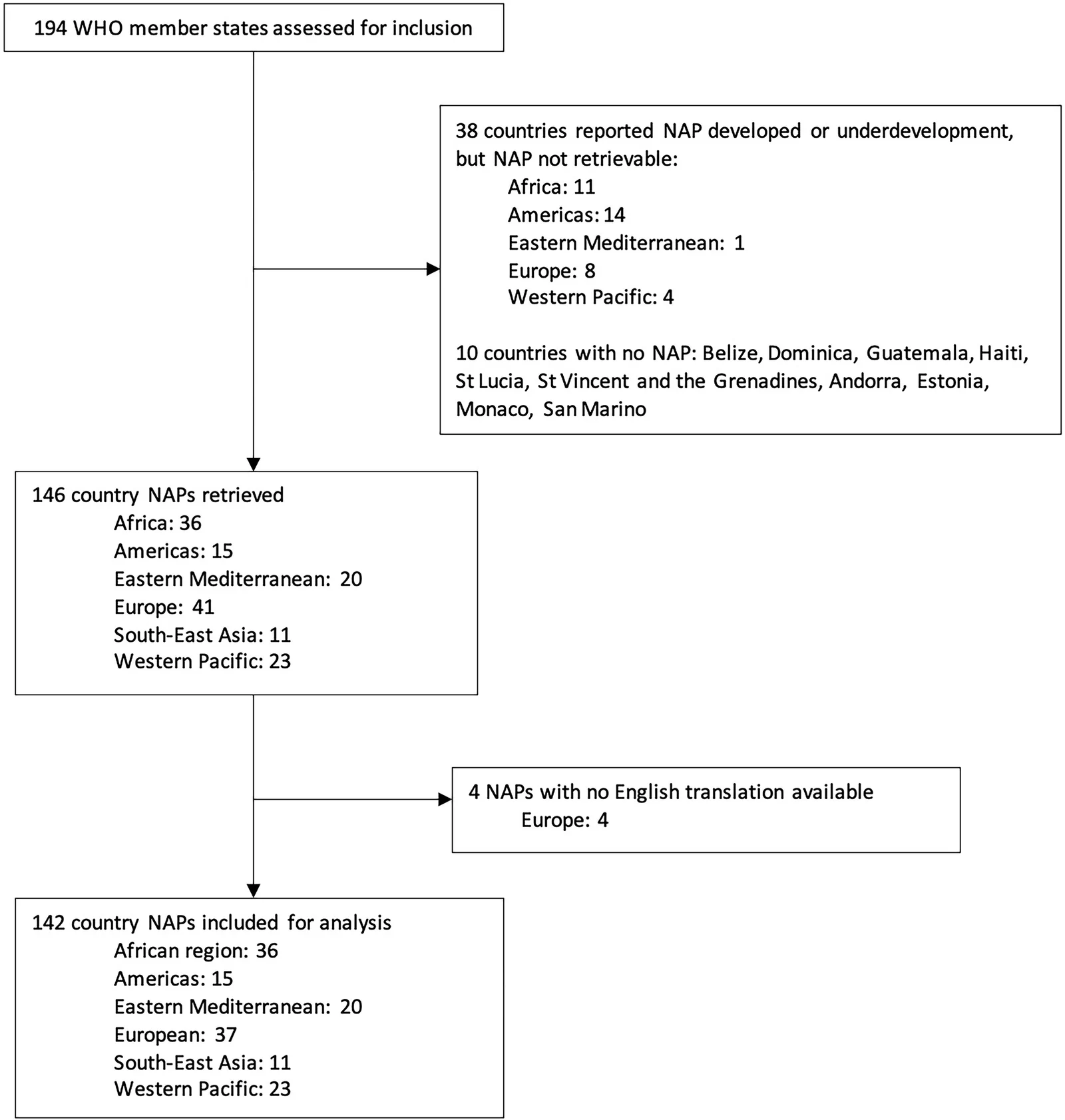
Preferred Reporting Items for Systematic Reviews and Meta-Analyses (PRISMA) diagram of search and retrieval of national action plans for inclusion in the analysis. Abbreviations: NAPs, national action plans; WHO, World Health Organization.

We identified NAPs for 146/194 (75.3%) WHO member states (Figure 1). Of the 48 WHO member states without a NAP, 3 (6.3%) were LICs, 12 (25.0%) were LMICs, 18 (37.5%) were UMICs, and 15 (31.3%) were HICs. Of 146 NAPs, 139 (95.2%) were from the WHO library and 7 (4.8%) were from other described sources. Four of 146 (2.7%) identified NAPs (Armenia, Croatia, Georgia, and Montenegro) were excluded because documents were not eligible for machine translation (Figure 2). One hundred forty-two of 146 (97.3%) NAPs were included for analysis, listed with source and dates covered in Supplementary Table 1.

### Review of NAPs for Evidence of Focus on Diagnostics

#### Diagnostic Modality

**Figure 3.**
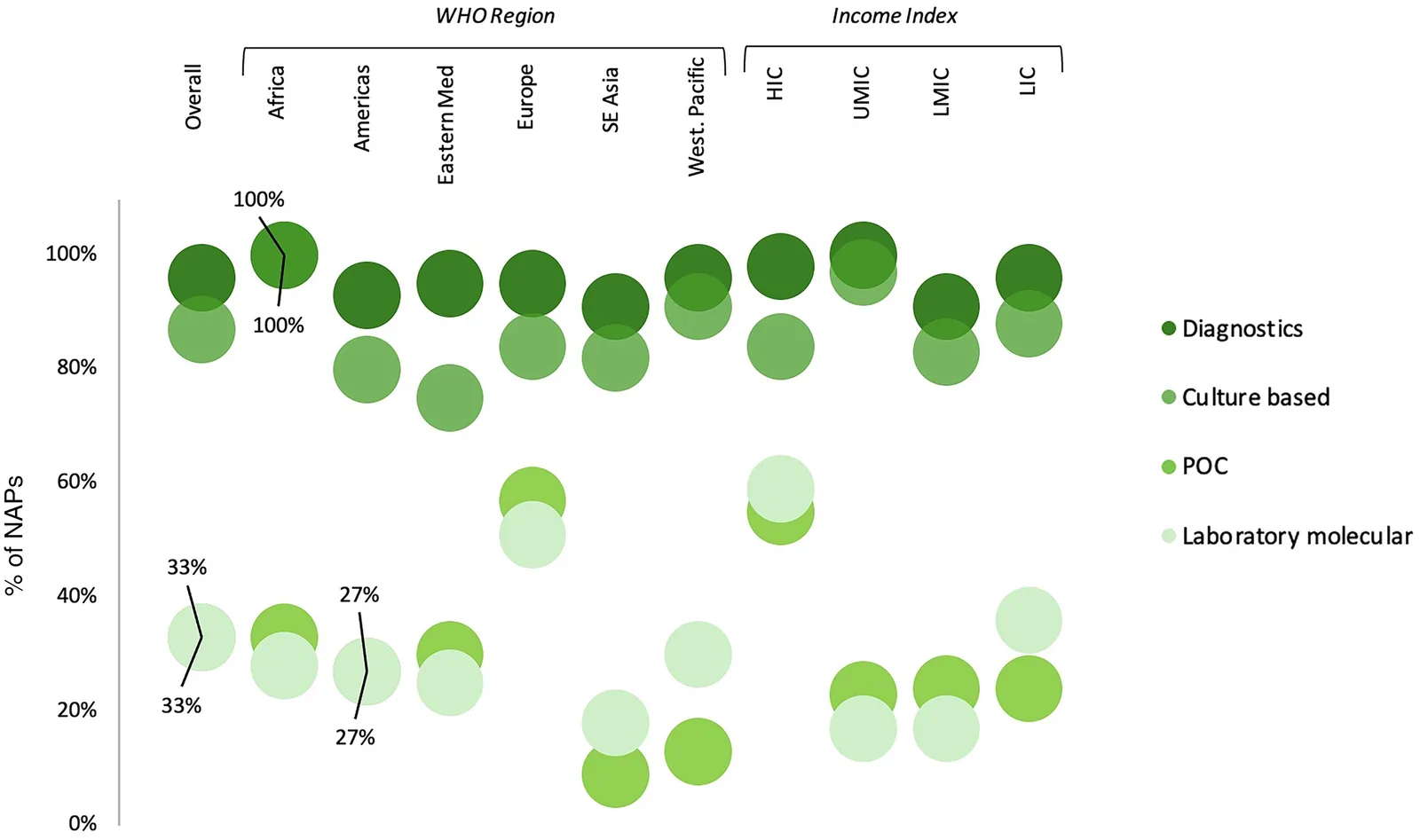
Bubble diagram demonstrating the percentage of national action plans that focus on diagnostics overall, and for each diagnostic modality, classified by World Health Organization region and World Bank income index. Abbreviations: Eastern Med, Eastern Mediterranean; HIC, high-income country; LIC, low-income country; LMIC, lower-middle-income country; NAPs, national action plans; POC, point-of-care; SE Asia, South-East Asia; UMIC, upper-middle-income country; WHO, World Health Organization.

Of 142 NAPs analyzed, 136 (95.8%) referenced diagnostics. The diagnostic modalities within NAPs varied across WHO region and World Bank income index (Figure 3). POC diagnostics were included most frequently in HIC NAPs (24/44 [54.5%]), and NAPs of the European Region (21/37 [56.8%]). POC diagnostics were included infrequently across NAPs of UMICs (7/30 [23.3%]), LMICs (10/42 [23.8%]), and LICs (6/25 [25%]) and least frequently in the region of South-East Asia (1/11 [9.1%]) (Supplementary Table 2; Supplementary Figure 1).

#### Diagnostics-Related Activities

**Figure 4.**
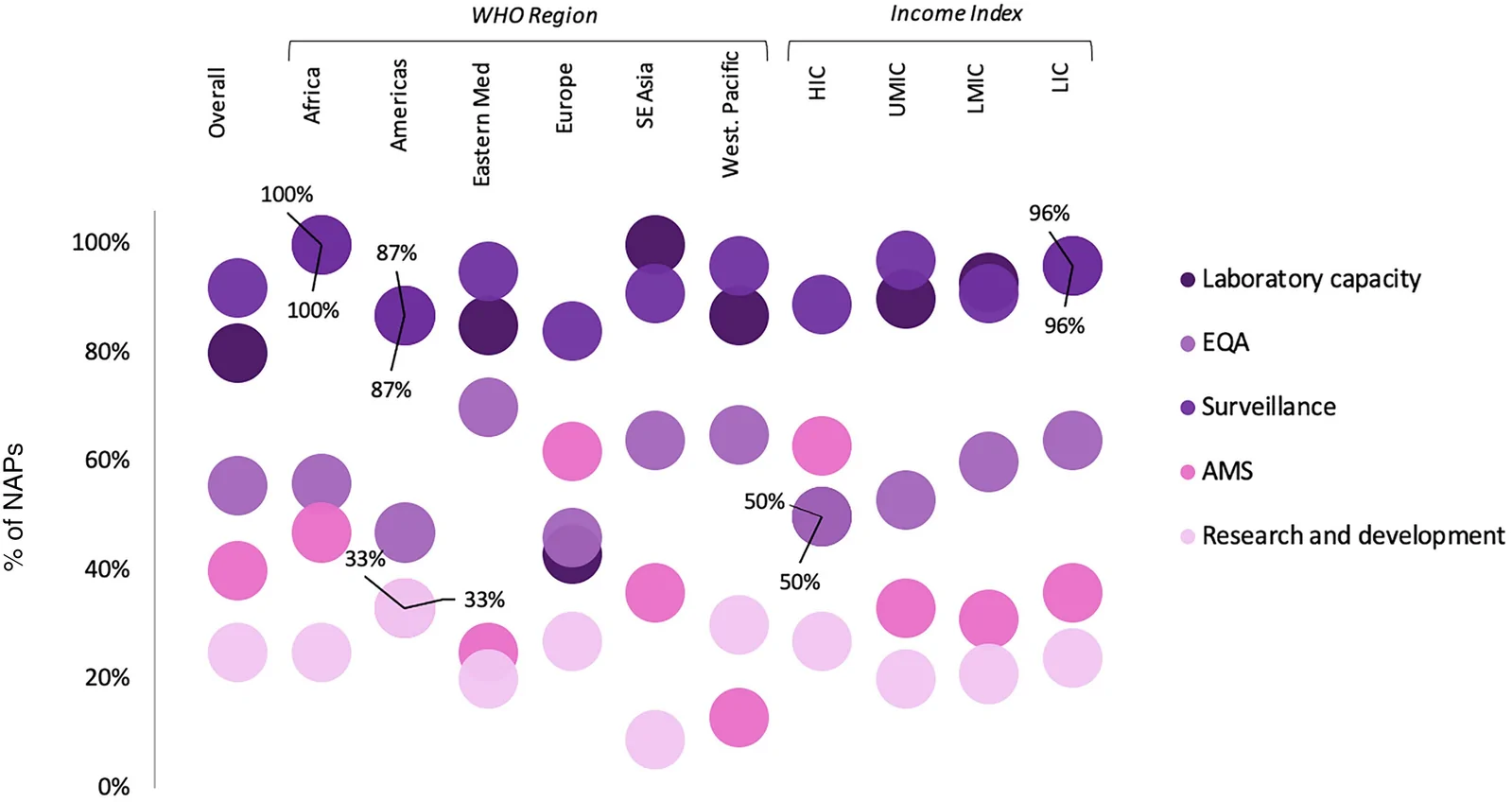
Bubble diagram demonstrating the percentage of national action plans that focus on diagnostics overall, and each diagnostic modality, classified by World Health Organization region and World Bank income index. Abbreviations: AMS, antimicrobial stewardship; Eastern Med, Eastern Mediterranean; EQA, external quality assessment; HIC, high-income country; LIC, low-income country; LMIC, lower-middle-income country; NAPs, National Action Plans; SE Asia, South-East Asia; UMIC, upper-middle-income country; WHO, World Health Organization.

Within NAPs, diagnostics were most frequently included in the context of enabling AMR surveillance (131/142 [92.3%]) and building laboratory capacity (113/142 [79.6%]), with highest rates for both activities in NAPs of the African Region (36/36 NAPs [100.0%]). Eighty of 142 (56.3%) NAPs included EQA for diagnostics. Focus on R&D occurred least commonly (36/142 [25.4%]), with highest rates of inclusion in NAPs of the Americas (5/15 [33.3%]) and those for HICs (12/44 [27.3%]) (Figure 4; Supplementary Table 2; Supplementary Figure 2).

#### Diagnostics-Specific KPIs

**Figure 5.**
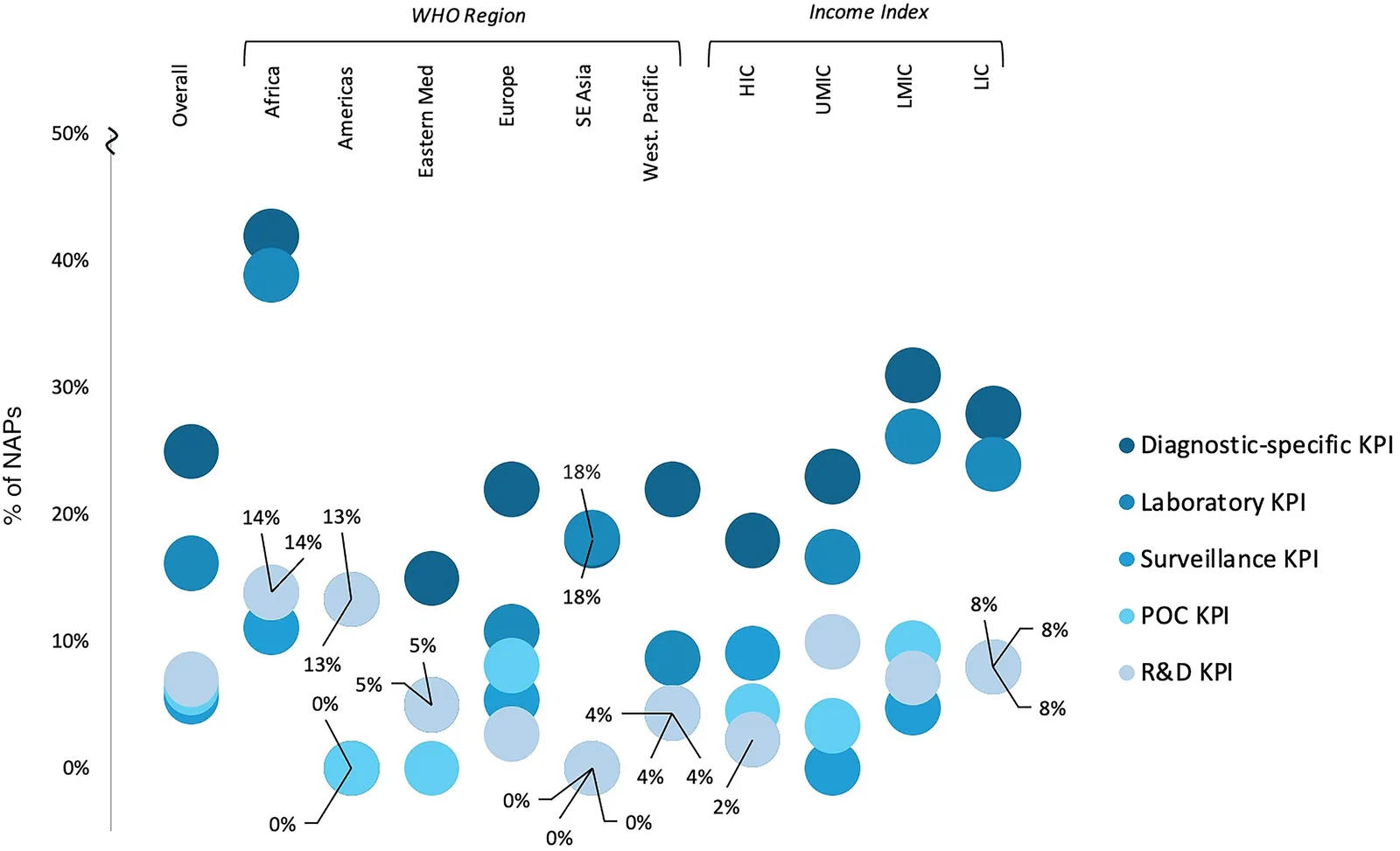
Bubble diagram demonstrating the percentage of national action plans that focus include a diagnostic-specific key performance indicator, and each key performance indicator theme, classified by World Health Organization region and World Bank income index. Abbreviations: Eastern Med, Eastern Mediterranean; HIC, high-income country; KPI, key performance indicator; LIC, low-income country; LMIC, lower-middle-income country; NAPs, national action plans; POC, point of care; R&D, research and development; SE Asia, South-East Asia; UMIC, upper-middle-income country; WHO, World Health Organization.

KPIs specific to diagnostics were present in 35/142 (24.6%) NAPs. Diagnostic KPIs were included most frequently in NAPs of the African Region (15/36 [41.7%]) and LMICs (13/42 [31.0%]), and least frequently in NAPs of the Americas (2/15 [13.3%]) and HICs (8/44 [18.2%]). Nine of 142 (6.3%) NAPs contained a KPI that focused on POC diagnostics. KPIs specific to POC diagnostics were most frequently included in NAPs of the African Region (5/36 [13.9%]) and LMICs (4/42 [9.5%]), and least frequently in NAPs of UMICs (1/30 [3.3%]) (Figure 5).

All NAPs that include diagnostic-specific KPIs are listed with examples, and classified by KPI theme, in Supplementary Tables 3 and 4.

#### Subregional Variation in the Americas

**Figure 6.**
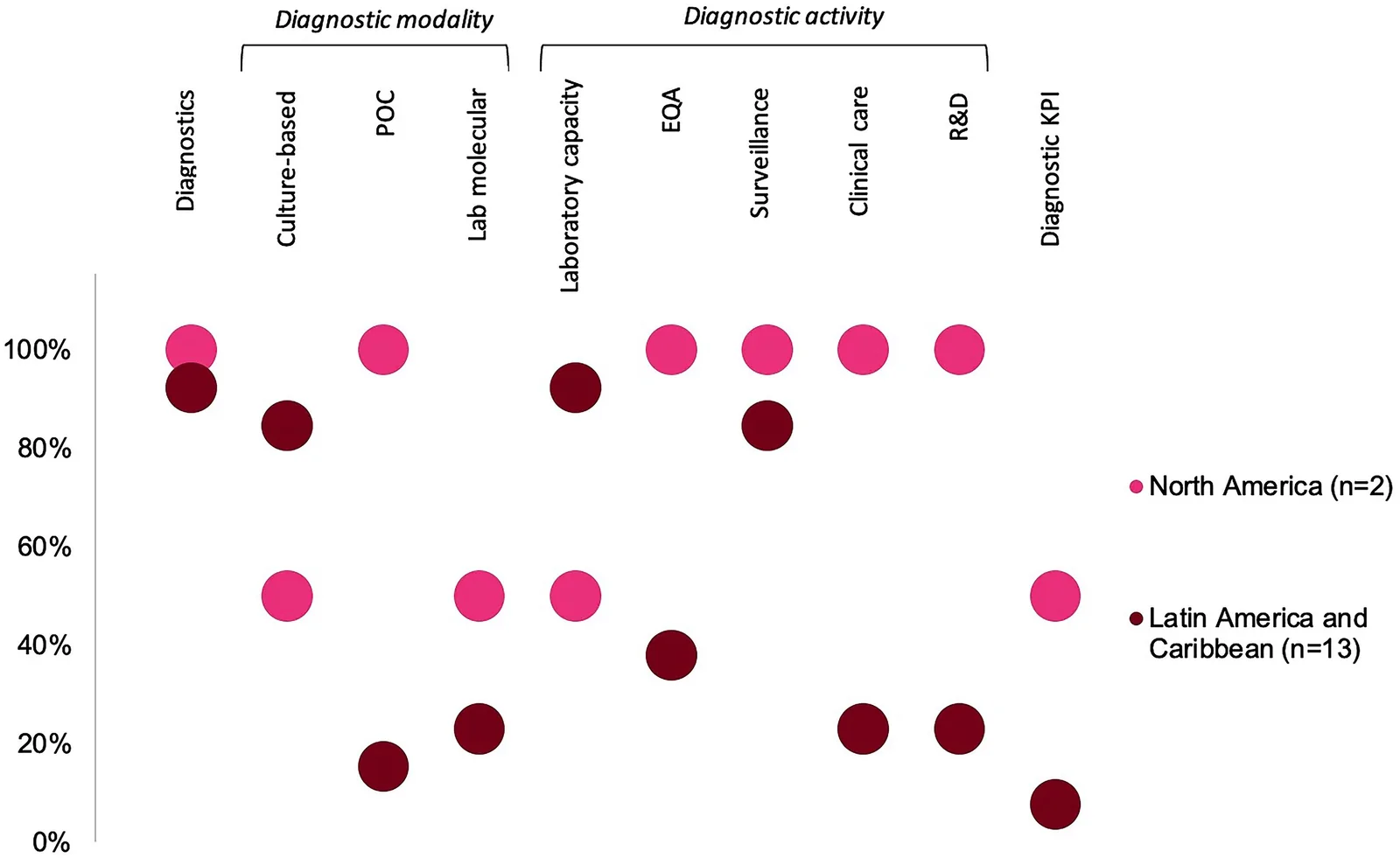
Bubble diagram demonstrating the percentage of national action plans within the Americas, classified according to subregions of North America (Canada and the United States) (n = 2) and Latin America and the Caribbean (LAC) (n = 13), that include focus on the diagnostic modalities and activities assessed, and include a diagnostic-specific key performance indicator. Both countries within the North America subregion are high-income countries (HICs), while in LAC, 3 are HICs, 8 are upper-middle-income countries, and 3 are lower-middle-income countries. Abbreviations: EQA, external quality assessment; KPI, key performance indicator; POC, point of care; R&D, research and development.

Diagnostics were referenced in 2/2 (100.0%) NAPs of North America and 12/13 (92.3%) NAPs of LAC. There was wide variation in the diagnostic modalities and activities included across these subregions (Figure 6). Diagnostic KPIs were included in 1/2 (50%) NAPs of North America and 1/13 (7.69%) NAPs of LAC.

### Qualitative Analysis

#### Diagnostic Modality

##### Culture-Based Diagnostics

Culture-based diagnostics were a key theme in NAPs of countries with limited diagnostic infrastructure, mostly in the African, Eastern Mediterranean, South-East Asia, and Western Pacific regions. For example, Niger acknowledges that few peripheral health facilities can perform AST with objectives to “implement standard operating procedures at sentinel sites for AST” and “purchase reference strains for culture, isolation, identification, and antibiograms.”

##### POC Diagnostics

POC diagnostics are frequently identified as tools for AMS. Algeria’s NAP highlights the lack of “rapid diagnostic tests for diagnosing bacterial infections in the community” and commits to improve access to “limit antibiotic prescription in outpatient clinics.” Oman’s NAP commits to “encourage the use of POC diagnostics to identify where antimicrobials are required,” citing rapid streptococcal antigen tests and rapid molecular assays for influenza as examples. Several NAPs (Austria, Ireland, Spain) commit to using C-reactive protein (CRP) POC tests to reduce inappropriate antibiotic prescribing.

Regional and lower-level health facilities emerged as priority sites for introduction of POC diagnostics due to limited laboratory access, with examples from HICs and LICs. Ghana’s NAP includes a target for 80% availability of rapid test kits at lower-level facilities by 2019. France recommends integrating POC tests into community and emergency care, with a particular focus on streptococcal RDTs.

Several NAPs included POC tests for rapid detection of bacterial resistance. Senegal commits to “reinforcing 30 health structures with GeneXpert capability” for diagnosis of multidrug-resistant tuberculosis. Argentina, Poland, Ireland, and New Zealand NAPs discuss rapid antigen diagnostics for detection of carbapenem-producing organisms, while the United States highlights limited research on POC testing for detecting AMR.

Few NAPs address fungal diagnostics; however, Italy’s NAP highlights a need for “POC implementation of simple molecular/antigen testing for *Pneumocystis* pneumonia and disseminated histoplasmosis,” as well as “rapid tests for resistance to azole drugs and echinocandins.”

##### Laboratory-Based Molecular Diagnostics

Laboratory-based molecular diagnostics were more likely to be included in NAPs of HICs. Some, such as Iceland, are actively introducing WGS for AMR testing while others (eg, Romania) suggest that capacity and cost analysis is required before implementation of WGS. Several LMICs (eg, Niger) propose implementing molecular diagnostics in parallel with culture-based testing as part of infrastructure development.

HICs with existing laboratory molecular capability trended to expanding current use and access, mostly to enhance and integrate One Health surveillance, but with varied detail. Germany will establish “an integrated molecular surveillance system for antibiotic-resistant pathogens of special importance,” the United Kingdom will use WGS to “enhance surveillance” alongside modeling and analytics, and Japan will “support pathogen analysis of AMR outbreaks utilizing WGS.”

#### Diagnostics Activities

##### Building Laboratory Capacity

Many NAPs recognize limited laboratory infrastructure as a barrier to effective AMR detection and control. This is apparent across regions and most marked for NAPs of LICs and LMICs. Sierra Leone states that “capacity for bacteriological culture and AST is limited by lack of skilled laboratory personnel, limited supply of reagents, and a weak supply chain system,” while Kenya notes that inadequate laboratory diagnostics have “contributed to indiscriminate use of antimicrobials.”

Several NAPs seek to improve availability of equipment and reagents: Benin aims to “equip hospital laboratories with basic equipment necessary for bacteriology with antibiogram capacity” and Eswatini targets renovation of 8 AST-capable laboratories. Countries with established laboratory networks focus on improving clinical access to diagnostics: Brazil intends all hospitals with intensive care units to have on-site AST, and Austria commits to 24-hour inpatient diagnostic access.

Workforce development is also prioritized. Tanzania’s NAP includes “training, mentorship, and supportive supervision for personnel on diagnostics and AST.” Many NAPs, including those of Chad, Gabon, Bhutan, Afghanistan, and Fiji, plan training workshops for laboratory staff, while Malaysia and Madagascar emphasize recruitment of laboratory staff and microbiologists.

##### EQA for Diagnostics

In Europe and the Americas, where diagnostic capacity is well-established, NAPs emphasize quality assurance and standardization of diagnostics. Common objectives include national AST standardization and participation in EQA schemes. Barbados’ NAP recommends “all national laboratories are involved in EQA programmes.” Several European NAPs (Iceland, Latvia, Poland) recommend compliance with European Committee on Antimicrobial Susceptibility Testing (EUCAST) methodology and submission of resistant strains to external reference laboratories; Serbia targets 90% laboratories implementing EUCAST methodology by 2022. The United Kingdom promotes international accreditation and EQA of laboratory diagnostics through the Global Antimicrobial Stewardship Accreditation Scheme.

##### AMR Surveillance at a Population Level

Some countries are yet to establish surveillance systems, for example Fiji, which aims to “establish a National One Health Reference Laboratory and integrated surveillance system by 2030.” Strengthening regional laboratory networks within countries is identified as an ongoing need; Malawi targets “100% of district laboratories implementing laboratory-based surveillance for AMR by 2022.”

HICs with established surveillance systems focus on modernization of diagnostic methods and data systems, including adoption of artificial intelligence and innovative digital tools. For example, Portugal aims to “maintain and improve the notification of alert and problem strains” within an electronic laboratory alert system.

##### Diagnostics for AMS at the Individual Patient Level

Most NAPs link diagnostics at the patient level to AMS. Nigeria aims to “bolster the National Diagnostic Stewardship Programme” using “clinical decision support systems.” Some NAPs propose diagnostics to justify clinicians’ decision-making regarding AMS: Finland will require a “diagnostic code on all antibiotic prescriptions”; Eswatini targets that “100% of eligible prescriptions are informed by culture and AST”; and Costa Rica promotes that “antimicrobial treatments are based on microbiological diagnostics and sensitivity tests,” although with limited implementation detail.

##### Research and Development

Around a quarter of NAPs reference R&D for diagnostics; however, most provide broad statements without detailed objectives or indicators in this area. For example, South Africa: “research into novel diagnostics including point-of-care testing”; Zimbabwe: “to invest research into quicker diagnostic tools”; and Canada: to “expand scientific knowledge base.” Some NAPs include specific financial or output targets: The United States commits to “support 10 new antibiotic resistance–related diagnostics projects by 2021, through funding or scientific or technical support”; Ghana allocates US$200 000 for novel POC diagnostics research; China aims to develop “5 to 10 new microbial diagnostic instruments, equipment, and reagents” by 2025; and Peru plans “at least 5 studies on diagnostic methods by 2021.”

## DISCUSSION

While most NAPs reference diagnostics, they vary greatly in the type, context, and level of commitment included. The emphasis on culture-based diagnostics, laboratory capacity building, and surveillance systems in LICs and LMICs reflects their early stage in diagnostic implementation. Willemsen and colleagues' 2022 review [5] identified that few African countries could perform adequate AST, justifying the focus of this region’s NAPs on these unmet needs. However, culture-based diagnostics are often impractical in LICs due to climate instability, unreliable electricity, and limited skilled personnel, which affect the reliability of AST [7, 17]. Indeed, huge discrepancies across contemporaneous susceptibility data submitted to different AMR surveillance platforms question data validity [2], and only half of NAPs commit to EQA for diagnostic accuracy. Deficient laboratory capacity and sociopolitical barriers have hindered AMR efforts in LMICs, particularly within LAC [18, 19], where availability of NAPs remains low.

POC diagnostics were underrepresented globally, appearing most frequently in HICs and European NAPs, suggesting that POC diagnostics are considered after more traditional testing is established. Prioritization by LICs of POC diagnostics, which require limited infrastructure and training, in parallel with strengthening laboratory capacity, could help to meet testing requirements. Cost-effectiveness of POC testing in sub-Saharan Africa has been demonstrated [20, 21], but LICs are disproportionately affected by supply issues [21]. Development of POC diagnostics largely occurs in HICs, under conditions suited to well-resourced health systems [22, 23]. Without addressing barriers to implementing POC diagnostics in low-income settings, including supply chain weaknesses, insufficient end-user knowledge, short shelf-lives, and prohibitive costs, benefits remain limited [7, 23–25]. Recent cuts in international aid have reshaped the global health funding landscape [26]. This provides an opportunity for LICs to develop sustainable diagnostic strategies fit for local contextual needs, through enabling policy frameworks that drive local diagnostic development [22, 27]. Notably, this study found that LICs and LMICs were more likely than HICs to include diagnostic-specific KPIs, suggesting that early strategic transformation is underway. However, very few of these KPIs target R&D; future NAPs must address this to ensure locally appropriate diagnostic innovation.

Few published studies on AMS interventions include diagnostics [28]; however, NAPs commonly recommend POC diagnostics like CRP testing to support AMS. Outcomes and cost-effectiveness of POC CRP testing studies vary, partly attributed to uncertainty in predicting future costs of AMR [8, 29, 30]. The AMR landscape has shifted dramatically since the GAP was published [31], and cost modeling could benefit from consideration of diagnostics’ wider impact on AMS [32]. The strongest focus on diagnostics for AMS was seen in HICs, where surveillance programs are already well-established. The focus on population-level AMR surveillance in LIC and LMICs is likely a positive response to WHO's calls under the GAP-AMR for expanded participation in the Global Antimicrobial Resistance and Use Surveillance System [10]; however, these NAPs now need to progress application of diagnostics from surveillance to informing AMS at the POC.

Diagnostics for fungal and parasitic infections were rarely included across NAPs. There has been widespread uptake of malaria RDTs in endemic regions, but increased antibiotic use among febrile children with negative-malaria RDTs has been observed, highlighting the need for broader diagnostic access [33]. Fungal infections are estimated to cause 2.5 million deaths annually [34], and the 2022 WHO Fungal Priority Pathogens List emphasizes the urgent need for improved fungal diagnostics and surveillance [35]. Despite this urgent appeal, evidence for implementation of fungal diagnostics remains scarce.

Previous analyses of NAPs have identified a lack of tangible objectives, with weak monitoring and evaluation plans [9, 36, 37]. The WHO's 2019 monitoring and evaluation framework for NAPs on AMR [38] does not set global targets but encourages countries to set national indicators, supported by a tailored monitoring and evaluation plan. Despite diagnostics being a core focus of 3 of the GAP-AMR objectives, only 25% NAPs have set KPIs in this area. This may stem from the lack of frameworks to value diagnostics, separate to their cost. This de-linkage of cost from value in AMR has already occurred in the United Kingdom for antimicrobials [39, 40], and a similar model could encourage increased funding for diagnostics. While most NAPs with KPIs include evaluation plans, only 5 countries to date (Bhutan, Fiji, Iceland, United Kingdom, United States) have published progress reports. Most NAPs included in this study are due for renewal: future iterations must include diagnostic-focused KPIs, with actionable evaluation plans and accountability for their progress, to ensure aspirational statements within NAPs are translated into real-world action against AMR.

### Limitations

This analysis is limited to publicly available, translatable NAPs. Although a rigorous search system was used to identify sources, we acknowledge there may be some published NAPs not included in our study. There is no existing framework for the assessment of prioritization of diagnostics within NAPs; themes were selected based on existing WHO policies and illustrative examples selected, but further thematic analysis is still needed. Patient and public involvement was not explored, which reflects a wider dearth of engagment with patients and public across AMR NAPs themselves. This study reviewed diagnostics for AMR in human health only; a One Health analysis of testing needs across animal and environmental sectors is now needed to guide AMR diagnostics innovation.

## Supplementary Material

ciag020_Supplementary_Data

## Supplementary Data

Supplementary materials are available at *Clinical Infectious Diseases* online. Consisting of data provided by the authors to benefit the reader, the posted materials are not copyedited and are the sole responsibility of the authors, so questions or comments should be addressed to the corresponding author.
