# Supplementary material for: Assessment of the Prioritization of Diagnostics in National Action Plans for Antimicrobial Resistance
Source: Clin Infect Dis. 2026 Apr 29;83(2):e228–36. doi: 10.1093/cid/ciag020 (PMC13537163; doi:10.1093/cid/ciag020)
Supplement: Supplementary file 1 [file ciag020_supplementary_data.docx]

Supplementary data

*Table 1:* List of National Action Plans (NAPs) included in the analysis, including source and dates covered, and classification by World Health Organisation (WHO) region and World Bank Income index classification.

| **WHO region** | **Country*** | **Income index^+^** | **Source** | **Years covered** |
| --- | --- | --- | --- | --- |
| **Africa** | Algeria | LMIC | WHO library | 2024-2028 |
|  | Benin | LMIC | WHO library | 2019-2024 |
|  | Burkina Faso | LIC | WHO library | 2018-2020 |
|  | Burundi | LIC | WHO library | 2020-2023 |
|  | Cabo Verde | LMIC | WHO library | 2018-2022 |
|  | Cameroon | LMIC | WHO library | 2021-2023 |
|  | Chad | LIC | WHO library | 2018-2021 |
|  | Comoros | LMIC | WHO library | 2022-2026 |
|  | Cote d’Ivoire | LMIC | WHO library | 2021-2025 |
|  | Democratic Republic of Congo | LIC | WHO library | 2018-2022 |
|  | Eritrea | LIC | WHO library | 2021-2025 |
|  | Eswatini | LMIC | WHO library | 2018-2022 |
|  | Ethiopia | LIC | WHO library | 2021-2025 |
|  | Gabon | UMIC | WHO library | 2019-2023 |
|  | Ghana | LMIC | WHO library | 2017-2021 |
|  | Guinea | LIC | WHO library | 2020-2024 |
|  | Guinea-Bissau | LIC | WHO library | 2023-2028 |
|  | Kenya | LMIC | WHO library | 2017-2022 |
|  | Liberia | LIC | WHO library | 2018-2022 |
|  | Madagascar | LIC | WHO library | 2019-2023 |
|  | Malawi | LIC | WHO library | 2017-2022 |
|  | Mali | LIC | WHO library | 2019-2023 |
|  | Mauritius | UMIC | WHO library | 2017-2021 |
|  | Mozambique | LIC | WHO library | 2019-2023 |
|  | Namibia | UMIC | WHO library | 2017-2020 |
|  | Niger | LIC | WHO library | 2025-2027 |
|  | Nigeria | LMIC | WHO library | 2024-2028 |
|  | Rwanda | LIC | WHO library | 2020-2024 |
|  | Senegal | LMIC | WHO library | 2018-2022 |
|  | Sierra Leone | LIC | WHO library | 2018-2022 |
|  | South Africa | UMIC | WHO library | 2017-2024 |
|  | Togo | LIC | WHO library | 2019-2023 |
|  | Uganda | LIC | WHO library | 2024-2029 |
|  | United Republic of Tanzania | LMIC | WHO library | 2023-2028 |
|  | Zambia | LIC | WHO library | 2017-2027 |
|  | Zimbabwe | LMIC | WHO library | 2017-2021 |
| **Americas** | Argentina | UMIC | WHO library | 2015 onwards |
|  | Barbados | HIC | WHO library | 2017-2022 |
|  | Brazil | UMIC | WHO library | 2018-2022 |
|  | Canada | HIC | WHO library | 2023-2028 |
|  | Chile | HIC | WHO library | 2021-2025 |
|  | Colombia | UMIC | WHO library | 2018 onwards |
|  | Costa Rica | UMIC | WHO library | 2018-2025 |
|  | Ecuador | UMIC | WHO library | 2019-2023 |
|  | El Salvador | LMIC | WHO library | 2022-2024 |
|  | Mexico | UMIC | Dof.gob.mx | 2013-2018 |
|  | Nicaragua | LMIC | Mimsa.gob | 2020-2024 |
|  | Paraguay | UMIC | WHO library | 2018-2020 |
|  | Peru | UMIC | WHO library | 2017-2021 |
|  | United States of America | HIC | WHO library | 2020-2025 |
|  | Uruguay | HIC | WHO library | 2018 onwards |
| **Eastern Mediterranean** | Afghanistan | LIC | WHO library | 2017-2021 |
|  | Bahrain | HIC | WHO library | 2019 onwards |
|  | Egypt | LMIC | WHO library | 2018-2022 |
|  | Iran | LMIC | WHO library | 2016-2021 |
|  | Iraq | UMIC | WHO library | 2018-2022 |
|  | Jordan | UMIC | WHO library | 2018-2022 |
|  | Kingdom of Saudi Arabia | HIC | WHO library | 2022-2025 |
|  | Kuwait | HIC | WHO library | 2022-2027 |
|  | Lebanon | LMIC | WHO library | 2019 onwards |
|  | Libya | UMIC | WHO library | 2019-2023 |
|  | Morocco | LMIC | WHO library | 2019-2022 |
|  | Oman | HIC | WHO library | 2020 onwards |
|  | Pakistan | LMIC | WHO library | 2017 onwards |
|  | Qatar | HIC | WHO library | 2024-2030 |
|  | Republic of Sudan | LIC | WHO library | 2018-2020 |
|  | Somalia | LIC | WHO library | 2022 onwards |
|  | Syria | LIC | WHO library | 2022 onwards |
|  | Tunisia | LMIC | WHO library | 2019-2023 |
|  | United Arab Emirates | HIC | WHO library | 2019-2023 |
|  | Yemen | LIC | WHO library | 2022-2026 |
| **Europe** | Albania | UMIC | WHO library | 2018-2020 |
|  | Austria | HIC | WHO library | 2021 onwards |
|  | Belgium | HIC | WHO library | 2020-2024 |
|  | Bulgaria | UMIC | WHO library | 2017-2021 |
|  | Cyprus | HIC | WHO library | 2012 onwards |
|  | Czech Republic | HIC | WHO library | 2019-2022 |
|  | Denmark | HIC | WHO library | 2017-2020 |
|  | Finland | HIC | WHO library | 2017-2021 |
|  | France | HIC | WHO library | 2022-2025 |
|  | Germany | HIC | WHO library | 2023-2030 |
|  | Greece | HIC | WHO library | 2019-2023 |
|  | Iceland | HIC | Stjornarradid.is | 2025-2029 |
|  | Ireland | HIC | WHO library | 2021-2025 |
|  | Italy | HIC | WHO library | 2022-2025 |
|  | Kyrgyzstan | LMIC | WHO library | 2022-2025 |
|  | Latvia | HIC | WHO library | 2019-2020 |
|  | Lithuania | HIC | WHO library | 2017-2021 |
|  | Luxembourg | HIC | WHO library | 2018-2022 |
|  | Malta | HIC | WHO library | 2020-2028 |
|  | Netherlands | HIC | WHO library | 2015-2019 |
|  | Norway | HIC | WHO library | 2015-2020 |
|  | Poland | HIC | WHO library | 2016-2020 |
|  | Portugal | HIC | WHO library | 2019-2023 |
|  | Republic of Macedonia | UMIC | WHO library | 2012-2016 |
|  | Republic of Moldova | UMIC | WHO library | 2019-2028 |
|  | Republic of Serbia | UMIC | WHO library | 2019-2021 |
|  | Romania | HIC | FAOLEX library | 2023-2030 |
|  | Russian Federation | UMIC | WHO library | 2019-2024 |
|  | Slovakia | HIC | WHO library | 2019-2021 |
|  | Slovenia | HIC | WHO library | 2019-2024 |
|  | Spain | HIC | WHO library | 2022-2024 |
|  | Sweden | HIC | WHO library | 2020-2023 |
|  | Switzerland | HIC | WHO library | 2015 onwards |
|  | Tajikistan | LMIC | WHO library | 2018 onwards |
|  | Turkmenistan | UMIC | WHO library | 2017-2025 |
|  | Ukraine | LMIC | FAOLEX library | 2019 onwards |
|  | United Kingdom | HIC | WHO library | 2024-2029 |
| **South-East Asia** | Bangladesh | LMIC | WHO library | 2017-2022 |
|  | Bhutan | LMIC | WHO library | 2018-2022 |
|  | Democratic republic of Timor-Leste | LMIC | WHO library | 2022-2026 |
|  | Democratic People’s Republic of Korea | LIC | WHO library | 2018-2020 |
|  | India | LMIC | WHO library | 2017-2021 |
|  | Indonesia | LMIC | WHO library | 2020-2024 |
|  | Maldives | UMIC | WHO library | 2017-2022 |
|  | Myanmar | LMIC | WHO library | 2017-2022 |
|  | Nepal | LMIC | WHO library | 2024-2028 |
|  | Sri Lanka | LMIC | WHO library | 2023-2028 |
|  | Thailand | UMIC | WHO library | 2017-2021 |
| **Western Pacific** | Australia | HIC | WHO library | 2020-2040 |
|  | Brunei Darussulam | HIC | WHO library | 2019-2023 |
|  | Cambodia | LMIC | WHO library | 2019-2023 |
|  | China | UMIC | WHO library | 2022-2025 |
|  | Cook Islands | Unclassified | WHO library | 2016 onwards |
|  | Federated states of Micronesia | LMIC | WHO library | 2019-2023 |
|  | Fiji | UMIC | WHO library | 2022-2025 |
|  | Japan | HIC | Mhlw.go.jp | 2023-2027 |
|  | Lao People’s Democratic Republic | LMIC | WHO library | 2019-2023 |
|  | Malaysia | UMIC | WHO library | 2022-2026 |
|  | Mongolia | LMIC | WHO library | 2022-2025 |
|  | Nauru | HIC | WHO library | 2021-2025 |
|  | New Zealand | HIC | WHO library | 2017-2020 |
|  | Papua New Guinea | LMIC | WHO library | 2019-2023 |
|  | Philippines | LMIC | WHO library | 2019-2023 |
|  | Republic of Korea | LMIC | WHO library | 2021-2025 |
|  | Republic of Marshall Islands | UMIC | WHO library | 2019-2023 |
|  | Samoa | LMIC | WHO library | 2023-2028 |
|  | Singapore | HIC | FAOLEX library | 2017 onwards |
|  | Solomon Islands | LMIC | WHO library | 2023-2026 |
|  | Tonga | UMIC | WHO library | 2017-2022 |
|  | Tuvalu | UMIC | WHO library | 2021-2025 |
|  | Vietnam | LMIC | WHO library | 2024-2025 |

*Classified by region, 36/142 (25%) NAPs included were from the African region, 15/142 (11%) from the Americas, 20/142 (14%) from the Eastern Mediterranean, 37/142 (26%) from Europe, 11/142 (8%) from South-East Asia, and 23/142 (16%) from Western Pacific.

^+^Classified by income index, 44/142 (31%) NAPs included were HICs, 30/ 142 (21%) UMICs, 42/142 (30%) LMICs, and 25/142 (18%) LICs with one NAP for an unclassified state (the Cook islands).

*WHO=World Health Organisation; HIC=high income country; UMIC=upper middle income country; LMIC=lower middle income country; LIC=lower income country; FAOLEX=Food and Agriculture Organisation database*

*Table 2:* Number (N) and percentage (N/n) of National Action Plans (NAPs) that include diagnostics, each diagnostic modality and diagnostic activity, classified by World Health Organisation (WHO) region and Income Index

| Type of diagnostic | Region | | | | | | | Income status* | | | |
| --- | --- | --- | --- | --- | --- | --- | --- | --- | --- | --- | --- |
|  | *Overall (n=142)* | *Africa (n=36)* | *Americas (n=15)* | *Eastern Med. (n=20)* | *Europe (n=37)* | *SE Asia (n=11)* | *Western Pacific (n=23)* | *HIC (n=44)* | *UMIC (n=30)* | *LMIC (n=42)* | *LIC (n=25)* |
| *Diagnostics* | 136 (96%) | 36 (100%) | 14 (93%) | 19 (95%) | 35 (95%) | 10 (91%) | 22 (96%) | 43 (98%) | 30 (100%) | 38 (91%) | 24 (96%) |
| *Culture based* | 124 (87%) | 36 (100%) | 12 (80%) | 15 (75%) | 31 (84%) | 9 (82%) | 21 (91%) | 37 (84%) | 29 (97%) | 35 (83%) | 22 (88%) |
| *POC* | 47 (33%) | 12 (33%) | 4 (27%) | 6 (30%) | 21 (57%) | 1 (9%) | 3 (13%) | 24 (55%) | 7 (23%) | 10 (24%) | 6 (24%) |
| *Laboratory molecular* | 47 (33%) | 10 (28%) | 4 (27%) | 5 (25%) | 19 (51%) | 2 (18%) | 7 (30%) | 26 (59%) | 5 (17%) | 7 (17%) | 9 (36%) |
| Diagnostic activity | | | | | | | | | | | |
| *Laboratory capacity* | 113 (80%) | 36 (100%) | 13 (87%) | 17 (85%) | 16 (43%) | 11 (100%) | 20 (87%) | 22 (50%) | 27 (90%) | 39 (93%) | 24 (96%) |
| *EQA* | 80 (56%) | 20 (56%) | 7 (47%) | 14 (70%) | 17 (46%) | 7 (64%) | 15 (65%) | 22 (50%) | 16 (53%) | 25 (60%) | 16 (64%) |
| *Surveillance* | 131 (92%) | 36 (100%) | 13 (87%) | 19 (95%) | 31 (84%) | 10 (91%) | 22 (96%) | 39 (89%) | 29 (97%) | 38 (91%) | 24 (96%) |
| *AMS* | 57 (40%) | 17 (47%) | 5 (33%) | 5 (25%) | 23 (62%) | 4 (36%) | 3 (13%) | 28 (63%) | 10 (33%) | 13 (31%) | 9 (36%) |
| *R&D* | 36 (25%) | 9 (25%) | 5 (33%) | 4 (20%) | 10 (27%) | 1 (9%) | 7 (30%) | 12 (27%) | 6 (20%) | 9 (21%) | 6 (24%) |

**excludes one NAP with an unclassified income index (Cook Islands)*

*WHO=World Health Organisation; POC=point of care; EQA=external quality assessment; AMS= antimicrobial stewardship; R&D= research and development; SE Asia=South-East Asia; HIC=high income country; UMIC=upper middle income country; LMIC=lower middle income country; LIC=lower income country*

*Figure 1:* Map charts demonstrating the global distribution of National Action Plans (NAPs) with inclusion of diagnostic modality: (A) culture-based diagnostics, (B) point-of-care, and (C) laboratory-based molecular diagnostics.

A

B

Included

Not included

C

*Figure 2:* Map charts demonstrating the global distribution of National Action Plans (NAPs) with inclusion of diagnostic-focused activities (A) building laboratory capacity, (B) external quality assessment of diagnostics, (C) surveillance at a population level, (D) diagnostics for antimicrobial stewardship (AMS) at a patient level and (E) research and development

A

B

C

Included

Not included

E

*Table 3:* List of country National Action Plan (NAPs) with diagnostic-specific key performance indicators (KPIs), classified by World Health Organisation (WHO) region and World Bank income status. KPIs are classified by 4 themes: laboratory, surveillance, point of care (POC) and research and development (R&D), and a quoted example from each NAP with a KPI is included.

| **WHO region** | **Country** | **Income status** | **Theme of KPI** | **Example*** |
| --- | --- | --- | --- | --- |
| Africa | Chad | LIC | Laboratory | Number of laboratories with updated standard procedure for germ identification and AST each year |
|  | Cote d'Ivoire | LMIC | Laboratory; POC | Raise awareness/organize an awareness campaign among stakeholders on the importance of microbiological tests in the rational use of antimicrobials (prospectus, flyers; distribution 1000 copies per year) |
|  | Eswatini | LMIC | Laboratory | Use of bacteriological diagnostics to inform antimicrobial prescribing: target for 100% of eligible prescriptions informed by culture and AST |
|  | Ethiopia | LIC | Laboratory; POC; R&D | Conduct research to identify rapid or best diagnostic methods and/or metrics to improve rational use of antimicrobials, aiming for 6 research studies published by 2025 |
|  | Ghana | LMIC | Laboratory; POC; R&D | Rapid test kits available in all lower level facilities and private sector practice: 80% availability for rapid test kits at lower levels by 2019 |
|  | Liberia | LIC | Laboratory | Conduct 5-days sessions to train laboratory technicians on the use of specialized diagnostics techniques and laboratory equipment: target of 261 within 5 years |
|  | Malawi | LIC | Laboratory; surveillance | Percentage of districts implementing laboratory based surveillance for AMR; target of 100% by 2022 |
|  | Mauritius | UMIC | Laboratory | Achieve accreditation for laboratories to process human, animal and environmental samples, specifically bacterial identification and AST: target of 4 laboratories accredited within 3 years. |
|  | Niger | LIC | Surveillance | Number of genomic AMR surveillance reports per year: target of one per year for every year of the NAP |
|  | Nigeria | LMIC | Laboratory; surveillance; R&D | Number of new diagnostics validated in Nigeria annually |
|  | Rwanda | LIC | Laboratory; POC; R&D | Establish a subcommittee that evaluates/ recommends appropriate/ affordable and accurate diagnostic tools: target 348 (at each health facility) in year 1. |
|  | Senegal | LMIC | Laboratory; POC | Implement and acquire the equipment for the antibiogram in 5 laboratories per year |
|  | Togo | LIC | Laboratory | 80% of laboratories are equipped with equipment and consumables that meet quality standards |
|  | United Republic of Tanzania | LMIC | Laboratory; surveillance | Number of laboratories included in the national AMR surveillance system in the human sector with the capacity to perform antimicrobial susceptibility testing and/or bacterial isolation. |
|  | Zimbabwe | LMIC | Laboratory; R&D | % of labs reporting susceptibility results out of all labs identified to provide diagnostic services |
| Americas | Peru | UMIC | R&D | Number of research studies on diagnostic methods: target of 5 by 2021 |
|  | United States of America | HIC | R&D | Support 10 new antibiotic resistance-related diagnostics projects across the U.S. Government by 2021, through funding or scientific or technical support. |
| Eastern Mediterranean | Egypt | LMIC | Laboratory | By 2022, 20% increase of labs in the national network that have capability to isolate pathogens and undertake antibiotic susceptibility testing in a quality manner |
|  | Kingdom of Saudi Arabia | HIC | Surveillance | Build capability for AMR molecular epidemiology at National AMR Laboratory at Weqaya to test up to 8,000 isolates" - target of 8,000 isolates/ year with plan for an annual report |
|  | Pakistan | LMIC | R&D | Number of research projects conducted for research into new vaccines and diagnostics |
| Europe | France | HIC | POC | Regarding streptococcal throat RDTs (2022-2023): number of RDT procedures carried out in pharmacies; number of boxes of RDTs ordered by physicians (GPs/paediatricians); |
|  | Iceland | HIC | Laboratory | The proportion of WGS on selected antibiotic-resistant bacteria carried out in Iceland in Iceland. - target for all MDR bacteria by 2029 |
|  | Italy | HIC | Surveillance | Proportion of microbiology laboratories involved in the surveillance system: target of 50% |
|  | Luxembourg | HIC | POC | Number of information and training tools distributed to prescribers and laboratories on the use of rapid diagnostic tests |
|  | Poland | HIC | Surveillance | An increase during the year in the rate of confirmation (10% compared to 2010) by the reference laboratory of the correctness of identification in hospital laboratories of dangerous resistance mechanisms in selected pathogenic bacterial species (carbapenem resistance in Enterobacteriaceae, Pseudomonas aeruginosa and Acinetobacter sp., vancomycin resistance in Enterococcus). |
|  | Republic of Moldova | UMIC | Laboratory; POC | Number of laboratories that have implemented rapid diagnostic methodologies annually |
|  | Serbia | UMIC | Laboratory; R&D | Implementation of the EUCAST14 methodology in all microbiology laboratories: target of 80% of laboratories with the EUCAST methodology introduced by 2020 and over 90% of laboratories with the EUCAST methodology introduced by 2022 |
|  | Turkmenistan | UMIC | Laboratory | Number of laboratories which perform AMS tests in line with the updated requirements |
| South-East Asia | Bhutan | LMIC | Laboratory | Microbiology testing facilities equipped: target of 15 by July 2019 |
|  | Sri Lanka | LMIC | Laboratory | Annual assessment on % of different levels of state sector & private hospitals with access to essential diagnostics without stockouts |
| Western Pacific | China | UMIC | R&D | One to three new antimicrobials should be developed and marketed, and five to ten new microbial diagnostic instruments, equipment, and reagents should be developed between 2022 and 2025 |
|  | Fiji | UMIC | Laboratory | Increase number of multisectoral microbiology laboratories conducting antibiogram tests by 10% in 2025 |
|  | Japan | HIC | Surveillance | The number of genomes accumulated in the genome database |
|  | Republic of Korea | LMIC | POC | Number of antibiotic-resistant bacteria rapid diagnostics-tools and therapeutics (act) developed |
|  | Vietnam | LMIC | Laboratory | Percentage of hospitals that perform microbiological techniques for diagnosis, treatment and monitoring of drug resistance: target of 40% by 2025 |

*Some NAPs included multiple KPIs across different diagnostic themes, but only one example is included per NAP

*WHO=World Health Organisation; KPI=key performance indicator; POC=point of care; R&D=research and development; HIC=high income country; UMIC=upper middle income country; LMIC=lower middle income country; LIC=lower income country; AST= antibiotic susceptibility testing; AMR=antimicrobial resistance; RDT=rapid diagnostic test; NAP=national action plan; WGS=whole genome sequencing; MDR=multi-drug resistant; EUCAST=European Committee on Antimicrobial Susceptibility Testing, AMS=antimicrobial susceptibility*

*Table 4:* Number (N) nand percentage (N/n) of National Action Plans (NAPs) that include diagnostic-specific key performance indicators (KPIs), and the theme of included KPIs, classified by World Health Organisation (WHO) region and World Bank income index

|  | Overall (n=142) | WHO Region | | | | | | Income index* | | | |
| --- | --- | --- | --- | --- | --- | --- | --- | --- | --- | --- | --- |
|  |  | Africa (n=36) | Americas (n=15) | Eastern Med (n=20) | Europe (n=37) | SE Asia (n=11) | West. Pacific (n=23) | HIC (n=44) | UMIC (n=31) | LMIC (n=42) | LIC (n=25) |
| Diagnostic KPI | | | | | | | | | | | |
| Included | 35 (25%) | 15 (42%) | 2 (13%) | 3 (15%) | 8 (22%) | 2 (18%) | 5 (22%) | 8 (18%) | 7 (23%) | 13 (31%) | 7 (28%) |
| Theme of KPI | | | | | | | | | | | |
| Laboratory | 23 (16%) | 14 (39%) | 0 | 1 (5%) | 4 (11%) | 2 (18%) | 2 (9%) | 1 (2%) | 5 (17%) | 11 (26%) | 6 (24%) |
| Surveillance | 8 (6%) | 4 (11%) | 0 | 1 (5%) | 2 (5%) | 0 | 1 (4%) | 4 (9%) | 0 | 2 (5%) | 2 (8%) |
| POC | 9 (6%) | 5 (14%) | 0 | 0 | 3 (8%) | 0 | 1 (4%) | 2 (5%) | 1 (3%) | 4 (10%) | 2 (8%) |
| R&D | 10 (7%) | 5 (14%) | 2 (13%) | 1 (5%) | 1 (3%) | 0 | 1 (4%) | 1 (2%) | 3 (10%) | 3 (7%) | 2 (8%) |

**excludes one NAP with an unclassified income index (Cook Islands)*

*WHO=World Health Organisation; KPI=key performance indicator, POC=point of care; R&D= research and development; HIC=high income country; UMIC=upper middle income country; LMIC=lower middle income country; LIC=lower income country*
